# Supplementary material for: Exploring the outcomes of non-surgical periodontal therapy in modulating periodontal parameters, renal function, and inflammatory biomarkers in chronic kidney disease patients with periodontitis
Source: PeerJ. 2025 May 29;13:e19492. doi: 10.7717/peerj.19492 (PMC12126971; doi:10.7717/peerj.19492)
Supplement: Supplemental Information 1 [file peerj-13-19492-s001.docx]

**SUPPLEMENTARY RESULTS**

*Supplementary Table 1.* *The mean, SD, SEM, and coefficient of variation (%) of the dental parameters (pre- and post-NSPT) of the CKD-P group.*

|  | PPD (Pre- NSPT) | PPD (Post- NSPT) | CAL (Pre- NSPT) | CAL (Post- NSPT) | GBI Score (Pre-NSPT) | GBl Score (Post- NSPT) | PS Score  (Pre-NSPT) | PS Score (Post- NSPT) |
| --- | --- | --- | --- | --- | --- | --- | --- | --- |
|  |  |  |  |  |  |  |  |  |
| Mean | 5.30 | 2.84 | 4.71 | 3.19 | 51.11 | 17.43 | 70.05 | 22.89 |
| Std. Deviation | 0.83 | 0.89 | 0.65 | 0.94 | 22.64 | 7.715 | 17.35 | 10.32 |
| Std. Error of Mean | 0.19 | 0.20 | 0.15 | 0.21 | 5.063 | 1.725 | 3.88 | 2.31 |
| Coefficient of variation (%) | 15.68 | 31.28 | 13.90 | 29.44 | 44.30 | 44.27 | 24.77 | 45.06 |
