# Supplementary material for: Exploring the outcomes of non-surgical periodontal therapy in modulating periodontal parameters, renal function, and inflammatory biomarkers in chronic kidney disease patients with periodontitis
Source: PeerJ. 2025 May 29;13:e19492. doi: 10.7717/peerj.19492 (PMC12126971; doi:10.7717/peerj.19492)
Supplement: Supplemental Information 2 [file peerj-13-19492-s002.docx]

**SUPPLEMENTARY RESULTS**

*Supplementary Table 2. The mean, SD, SEM, and coefficient of variation (%) of the dental parameters (pre- and post-NSPT) of the P group.*

|  | PPD (Pre- NSPT) | PPD (Post- NSPT) | CAL (Pre- NSPT) | CAL (Post- NSPT) | GBI Score (Pre-NSPT) | GBl Score (Post- NSPT) | PS Score (Pre-NSPT) | PS Score (Post- NSPT) |
| --- | --- | --- | --- | --- | --- | --- | --- | --- |
|  |  |  |  |  |  |  |  |  |
| Mean | 5.01 | 2.26 | 3.85 | 2.30 | 45.00 | 19.04 | 54.33 | 19.86 |
| Std. Deviation | 0.68 | 0.74 | 2.01 | 1.21 | 21.54 | 8.17 | 26.24 | 8.33 |
| Std. Error of Mean | 0.15 | 0.16 | 0.45 | 0.27 | 4.82 | 1.83 | 5.87 | 1.86 |
| Coefficient of variation (%) | 13.59 | 32.69 | 52.18 | 52.61 | 47.85 | 42.92 | 48.30 | 41.93 |
